# Reliability and Mechanical Properties of Materials Recycled from Multilayer Flexible Packages

**DOI:** 10.3390/ma13183992

**Published:** 2020-09-09

**Authors:** Natividad Antón, Álvaro González-Fernández, Alberto Villarino

**Affiliations:** 1 Department of Construction and Agronomy, Materials Science and Metallurgical Engineering Area, High Polytechnic School of Zamora, University of Salamanca, Avda. Requejo 33, 49022 Zamora, Spain; nanton@usal.es (N.A.); alvarogf3pa@icloud.com (Á.G.-F.); 2 Department of Construction and Agronomy, Construction Engineering Area, High Polytechnic School of Ávila, University of Salamanca, Hornos Caleros, 50, 05003 Ávila, Spain

**Keywords:** material recycling, reliability, mechanical properties materials

## Abstract

Present work proposes a recycling form of multilayer food packages to avoid their incineration, landfill of wastes or re-using of their individual components. The manufacturing process of the material consists of binding several sheets by the non-simultaneous application of temperature and pressure, these being bound by the fusion of the polyethylene, without any other adhesives. The influence of elected variables (temperature, pressure, time and number of sheets) on the mechanical properties is determined. Bending tests in three points were carried out, with the purpose of obtaining flexural strength and flexural modulus. Finally, the reliability was determined through the method of Weibull using different failure estimators for the most appropriate materials (relative to flexural strength and porosity values). Light microscopy to obtain information about defectology before and after tests was used. The results obtained by this type of material are very similar to those presented by wood-based materials and present good reliability from the strength point of view.

## 1. Introduction

**Figure 1:**
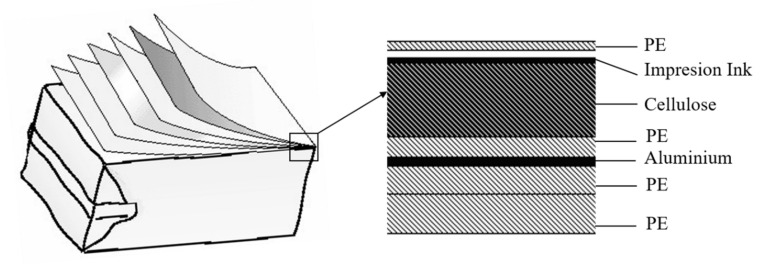
Aseptic cardboards (UHT products).

The multilayer packages (or aseptic cardboards, Tetra Pak^®^, Pully, Switzerland) used in the food industry have their origins in 1951 [1,2]. Initially, they demonstrated a great capacity to protect and conserve the foods due to an excellent design of the layers (polyethylene, aluminium and cardboard) and their properties [3]. The polyethylene (PE) layers provide liquid tightness and adhesion with the other components. While, the cardboard (cellulose) provides stiffness and the aluminium protects from the environment (Figure 1). International Food Institute in 1989 proclaimed this technology as one of the most important discoveries in the alimentary industry.

One of the most important points, in production or design, is the Life Cycle Assessment or LCA [4,5,6]. The environmental influences of the multilayer packaging materials components that could cause in incineration or landfill wastes are very important [7] Different problems are generated by wastes of multilayer packaging, as many as its basic components, because its consumption or useful life is short and produces a lot of waste. Below is shown some aspects related to the different components of Tetra Pak^®^ that influence on the package LCA.

Aluminium is produced from bauxite and is a non-renewable (as all the minerals and respective metals) resource [8,9]. It is necessary determine its recycling quota [10]. Aluminium production is an industrial process that consumes a great amount of energy [8,9,10,11]. The process has an important environmental impact, due to several tons of mineral wastes [12]. Now, the recycling of aluminium has become indispensable and saves 95% of the necessary energy used in the conversion of the bauxite. Aluminium is one of the most widely used materials, and the technology associated with its recycling is extensive. This makes the studies more and more specific: The direct recovery of aluminium scraps by hot-pressing forging [13], or the use of low-temperature molten salt electrolysis [14]. Although the recycling process of the aluminium by re-melting of cans and cuttings is very rentable compared with hydrometallurgical process [10], this is not so evident in the case of separation of layers of aluminium of the food packages, because it is necessary to try a great volume of wastes to procure a minimum amount of metal. Adding to this, the formation of aluminium carbides [15] could pollute the obtained alloys.

Another one of the components of the multilayer material is polyethylene (PE). It belongs to the family of the thermoplastics and according to its branching grade, it is classified as Low Density PE, (from 0.941 to 0.925 g/cm^3^) rather than Medium Density (from 0.926 to 0.940 g/cm^3^) and High-Density (from 0.941 to 0.965 g/cm^3^). There are studies about recycling of PE of different origins, but the main problem is its degradation in re-processing [16]. Its melting temperature is around 130 °C and presents adhesive properties [17,18]. The adhesives present several advantages with respect to other methods of assembling materials: With a uniform distribution of tensions without any distortion of the substrate, they also allow a sealing union between different materials. The layers of PE, which are the finest possible, guarantee perfect union in the packages. The environmental impact of this material is the same as in the petroleum industry, one of the most polluting sectors.

The last component of the multilayer package is the cardboard, as a variant of the paper (cellulose), it is composed of several layers of cellulose, those which overlapped and combined, give stiffness and strength properties. The paper (cellulose) is one of the most used materials [19]. A great part of the paper is derived from the pulp of trees and other vegetable fibres. Although their recycling is very easy, in fact, paper is recycled for the most part. The demand for paper (or cardboard) is growing, and this forces the manufacturing of great quantities of cellulose paste, which cause the pruning of millions of trees [7].

One of the first stages inside the new recycling technology is separation [20]. The most rational and economic way to classify wastes is realizing this at home [21]. The multilayer packages, and in general all those that are made of cardboard, are recoverable as a cellulose resource, but their separation from the rest of the Solid Urban Wastes is necessary [22]. There are very diverse systems to collect packages to recycle [23], but all require an active participation of the consumer, adapting to each population and management infrastructure of wastes.

There are many methods to recycling these type of packages. The most habitual way is employing the Hydrapulper [24], where the basic components are recycled as separate materials [25]. Another form is recycling the package as a group [26], making a pulp of composite material used like urban furniture. The raw material in this method is processing as pulp, with crushing, warm pressing and cooling, to produce agglomerate sheet. There are hardly any references respect properties the final parts (strength, reliability, etc.). Another method is chemical separation, to separate the polyethylene in dissolution [27]. It can also recover the aluminium for combustion (Corenso process [28]), where the final product is cardboard for tubes and packings, and PE and the aluminium are integrated into the energy cycle of the process. The PE can be recycled by extrusion [16], co-extrusion [29], etc. There are studies investigating the possibility of valuing these packages directly by pyrolysis [30]. On the other hand, there are also studies in which aluminum-polluted polyethylene is recovered (several types of packages) to obtain nanoparticles [31]. In all cases, the process leads to the destruction of susceptible raw material without separation or with partial separation.

Another important point to evaluate the life-cycle of a waste is the decomposition time. This factor is one of the most important to evaluate the environmental impact of the different wastes [4,5,6,10]. Not all the wastes biodegrade quickly. These processes can be very slow and produce undesirable effects, which makes its recovery and new usage more convenient. When materials degrade, they lose the positive effect that their controlled decomposition could be obtained [6] or of their recycling. To this, it would be necessary to add the maintenance of landfilling occupy space, and which can cause water contamination. 

The three points bending test is one of the most habitual to determine the flexural strength for main materials [32]. It is widely employed due to its simplicity and the quantity of information that gives. In this work, it is used as the key property for the determination of the best processing conditions. In addition, it is widely used to determine flexural strength of woods and agglomerates [33]. This property is good to determine the reliability of materials. Diverse studies exist on composites materials based on wood and plastic or WPC (Wood Plastic Composites) [34,35]. This type of composite material could be obtained by hot pressing, extrusion or injection moulding, reaching three point flexural strengths between 20 and 40 MPa [36,37,38].

In composite materials, the random distribution of the defects could be adjusted to considerations of statistic type. The Weibull distribution is broadly utilized to measure the reliability of materials, ceramic matrix composites [39], and polymeric composites [40]; for example, epoxy-carbon [41], composite materials based on Portland clinker [42], as well as compound materials for dental use [43] are used to determine the dispersion in the mechanical results. The probability of failure of the mechanical parts is well adjusted to this distribution, and the mechanical properties of a composite material can be expressed through the parameters of the following equation, considering the same size for all the specimens:

(1) $$\mathrm{Ln}\cdot \mathrm{Ln}\frac{1}{\left(1-F(t)\right)}=\beta \mathrm{Ln}\sigma_{\max.}-\frac{{\mathrm{Ln}}_{0}^{\beta }}{V_{E}}$$

the failure probability is related with applied stress through this lineal equation, where β is the line slope and gives an idea of dispersion of the mechanical results for each material, and α is the scale factor represented as Ln σ^β^_0_/V_E_. The values α and β can be graphically determined by Equation (1). This expression fits a straight line (y = ax − b), where slope represents the reliability of the material with respect to a key property.

To evaluate the strength properties of a composite material, average strength and Weibull modulus are fundamental. It is more probable to obtain more homogeneous results and higher modules of reliability with specimens of little size, due to higher probability of finding critical defects in big parts. However, it is possible to extrapolate from a size to another. Different failure estimators (Fj) can be used [44]. Although the Weibull method to characterize the reliability of a material is broadly utilized in diverse fields, there are no studies applied to wooden materials.

The fundamental objective of this study has been to evaluate the recycling of the multilayer packages (recyclability), without destroying this laminate distribution and its mechanical properties. Because the processing of the materials is based on the adhesion capacity of polyethylene (PE) and the interface of bonding is the most critical zone with a higher probability of failure, this work presents data of reliability and microstructural characterization that could be interesting and applicable in various scientific fields.

Currently, a great concern exists about the environmental damages caused by the wastes. This supposes to generate new cyclic process (re-feeding material into the process) and usage habits with the purpose of avoiding the waste of raw materials and energy, in a word: re-using. Then, this work thinks about the alternative of recycling multilayer packages, avoiding the separation of its components, the re-melting or its incineration. This study attempts to present data on flexural strength without destroying the multilayer structure, which may have advantages over the more common use as agglomerate board or panel, since strength would be improved for the same use. In addition, the reliability of the material will be seen with respect to two key properties such as flexural strength and porosity.

## 2. Materials and Methods 

The first step was the election of the manufacturing variables (temperature, pressure, time, and number of sheets) and the levels (two) to generate the factorial design, according to previous manufacturing tests in this work. Parallel, the different stages of the manufacturing process were described as follows:

(1) Washing of Aseptic type Tetra Brick^®^, to avoid possible problems in the polyethylene adhesion, achieve a better surface homogeneity and minimize defects to increase reliability. Many types of Tetra Pak (multilayer packages) are commercialized, so those with similar specifications were chosen to homogenize the quality of the raw material. In this case, milk packages of GAZA.S.L. (Ganaderos de Zamora S.L. Zamora, Castile and Leon. Spain) were selected.
(2) Cutting of the packages by the edges obtaining identical dimensions of multilayer material (sheet).
(3) Placing the sheets symmetrically: 6 or 10 (previous tests indicate that more sheets could not be glued in present conditions), to force the contact between PE–PE layers of the two central sheets.

The elected temperatures were higher than polyethylene melting point to reach its adhesive behaviour. This temperature is around 115–140 °C [45], but in this temperature range, the phenomena of adhesion is little. The moulding process of polyethylene is normally carried out at temperatures of the order of 180–190 °C [46,47]; the final elected temperatures were T_1_ = 170 °C and T_2_ = 190 °C. On the other hand, temperatures higher to those could unnecessarily degrade the cellulose and polyethylene, so 190 °C was the higher limit of temperatures for this type of process.

**Figure 2:**
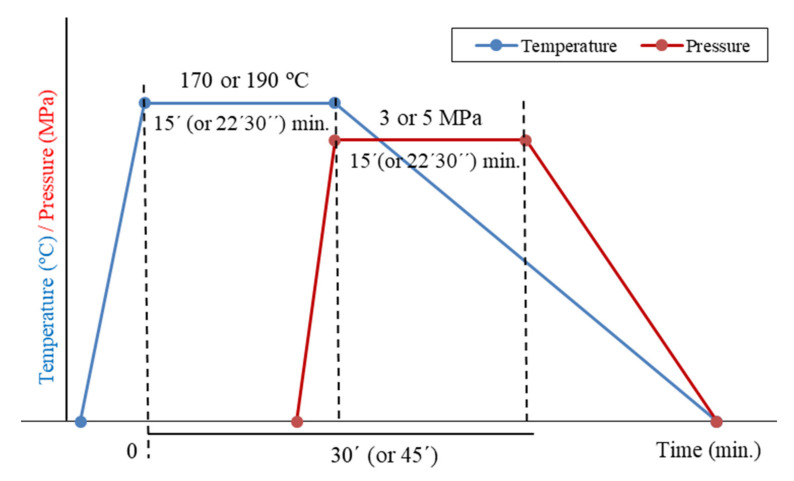
Basic manufacturing outline.

The basic manufacturing outline of the samples is reflected in Figure 2. The time was the minimum below which adhesion takes place. This time should not be very big, avoiding any unneeded energy costs. Adhesion did not take place for maintenance time below 30 min, based on previous tests. This way, the levels were t_1_ = 30 min. and t_2_ = 45 min. For the election of the pressures, the adhesion between sheets is not produced (previous pre-tests in this work) at low pressures and the intermediate sheets slipped at high pressures with PE losses. As a consequence, the elected pressures were approximately 3 MPa and 5 MPa. Finally, the chosen numbers of sheets were 6 and 10. Relative orientation of multiple sheets was also considered in the initial tests, but this variable was discarded because the cellulose fibers had no orientation.

Once the levels were elected, the applied factorial design was 2^3^ or 2^(4−1)^ (considering that the number of sheets would depend on the rest of variables), obtaining eight groups of conditions (Table 1). Three samples for each group, to obtain average values of flexural strength and flexural modulus, were manufactured.

**Figure 3:**
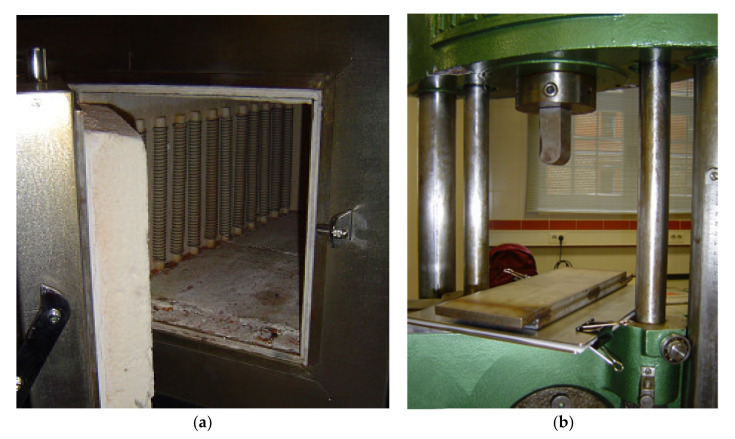
(**a**) Furnace and (**b**) pressing machine 10 Tn. Max.

**Figure 4:**
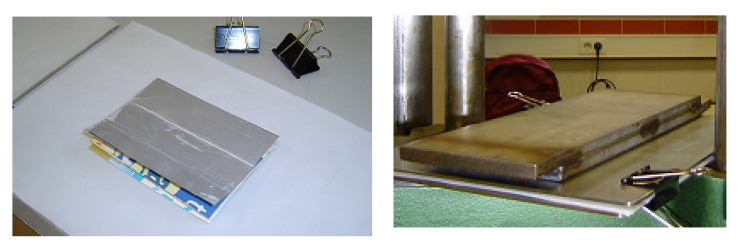

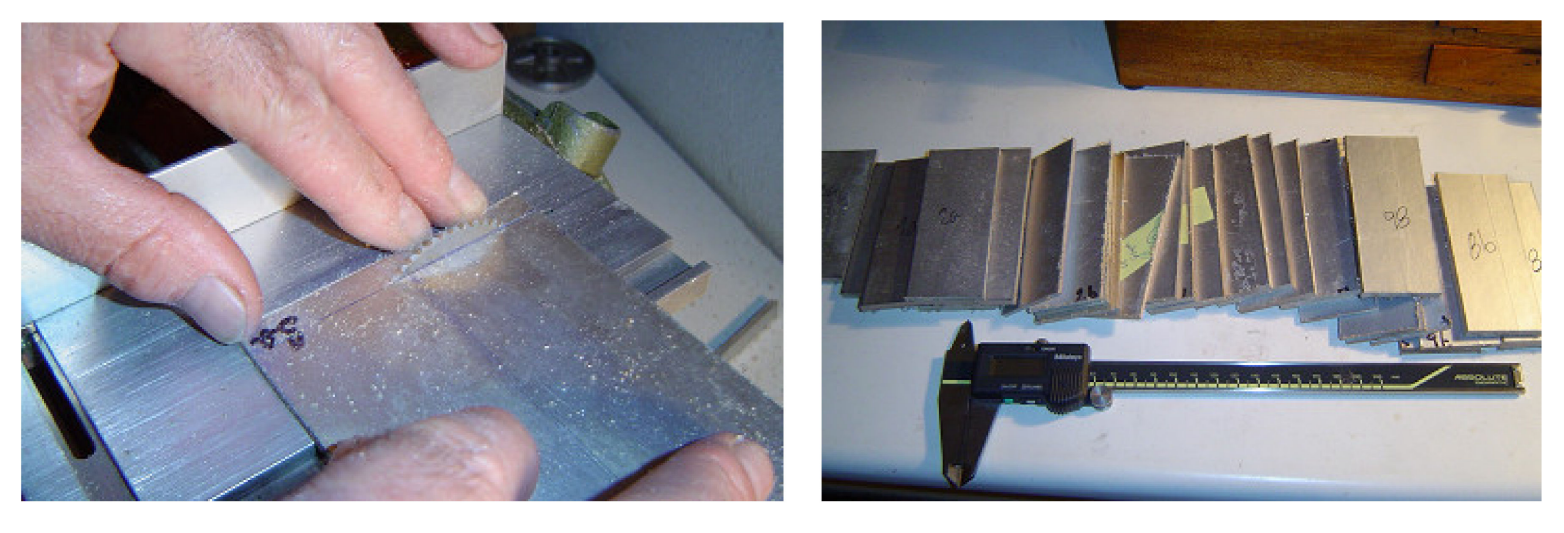
Operations performed to obtain the parts (boards of laminate composite material).

After selection and cleaning operations of the raw material, an operation of heating of the samples in a furnace (Chesa Model 1200 °C max. (Madrid, Spain)—Figure 3a) was made at the temperatures and elected times. Next, the boards (85 mm × 115 mm) were pressed at elected pressures, corresponding to the different groups (Table 1). In the warm pressing operation (Universal Pressing Machine Model Nestor 10 Tn. max. (Madrid, Spain)—Figure 3b), teflon sheets were used to avoid the adhesion between PE and the steel-pressing parts. Figure 4 represents the group of operations to obtain the test parts.

Weight measures (before and after conformation process) were made to check any appreciable variations. Afterwards, a cutting operation was carried out as well as elimination of burs or imperfections to reach final dimensions (40 mm × 100 mm approximately). The final polishing was made to eliminate surface defect and avoid notch effect.

The dimensions of the board samples are the thickness and the width, and long is was imposed by the separation between supports, according to UNE-EN-408 [48], UNE-EN-384 [49], and UNE-EN-1194 [50] relatives to the structural wood and adapted for the tests. The result was some boards of laminate composite material with an aspect of agglomerate sheets and getting bonded without adhesives (Figure 4). Carrying out a non-simultaneous cycle of temperature and pressure, there are not polyethylene losses of the boards, guaranteeing the adhesion that is required.

To determine the density, dimensions were measured with a Mitutoyo Model CD-6’’C caliber (±0.01 g, Kawasaki, Japan) and the weight on a KERN Model ALS 220-4N precision balance (±0.0001 g, Balingen, Germany).

As the previous step to the bending tests, the characterization of samples-boards was carried out; determining its density, compacting grade and porosity (through its weight and dimensions). To measure the density and derivative variables, the weight was measured with a precision of ±0.001 g and for the dimensions the precision was ±0.01 mm. To calculate the porosity, it is necessary to keep in mind the calculated density of the single package (0.981 g/cm^3^) and the density of the obtained parts. Afterwards, bending tests were carried out for the eight processing conditions.

**Figure 5:**
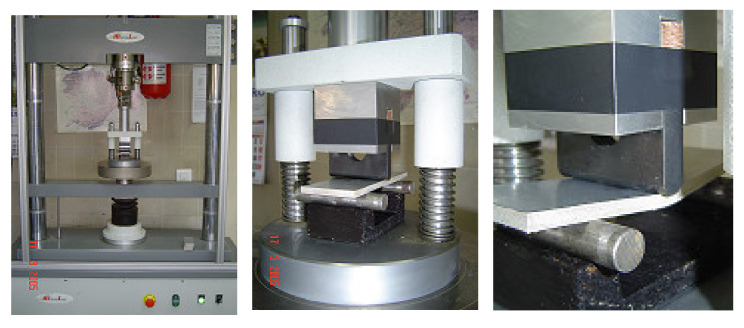
Microtest Model EM 1/200FRM, with transducer of 5 kN employed.

The three point bending tests were carried out in a machine Microtest Model EM 1/200FRM (Madrid, Spain), with a transducer of 5 kN (Figure 5), using computerized data. The speed of load application was 1 mm/min. With the generated data (load and displacement), graphs were obtained to determine flexural strength and flexural module.

Once the different flexural strengths were determined, the two most resistant (of 6 and 10 sheets) materials were chosen. In each of these two groups, 20 tests were obtained to carry out the reliability through the determination of its Weibull modulus.

The section of the samples was characterized (before and after fabrication process), with the purpose of determining the appearance and common defectology. After the fabrication stage and the realisation of flexural tests, a microstructural study was carried out on the most significant parts using an optical microscope ZEISS Axiovert 100A (Jena, Germany) and a connected digital camera MOTIC (Hong Kong, China). In some cases, the different images were assembled to give a more general image of the parts, the different types of breaks and their defectology. The parts were polished to be observed under a microscope, using a 1000 SiC sandpaper and trying to be as smooth as possible.

## 3. Results and Discussion

**Figure 6:**
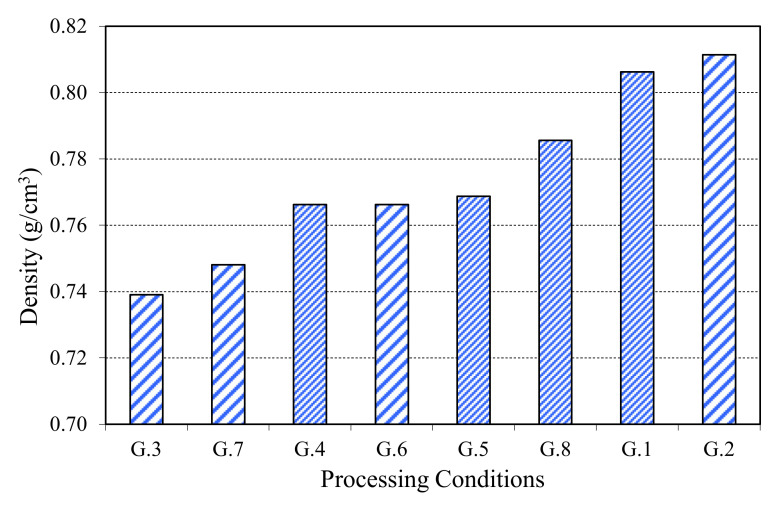
Density of the materials (in growing order).

Figure 6 represents the results of density (in growing order) of different obtained samples. The average density obtained shows that the groups G1 and G2 (Figure 4) have the maximum values of density, in front of the other ones. The combined effect of the pressure and temperature produces some denser pieces. In a parallel way, the compression grade (calculated by variance of the thickness before and after the process) for the parts of 10 sheets oscillated between 11 and 15%; while in the samples with six sheets, the variance of thickness was around 2 and 7%. The compression grade fits the next equations: for 10 sheets (y = −20.674x + 100.15) and for 6 sheets (y = −36.061x + 100.25). The groups G1 and G2 also showed the biggest compression grades. The adhesive effect of the polyethylene was more effective with the lowest number of sheets, due to the biggest effect in the pressure and temperature and against the lowest number of interfaces.

**Figure 7:**
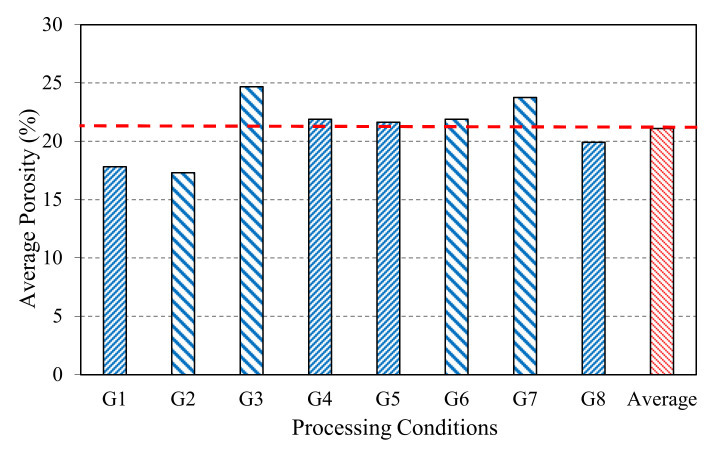
Average grade of porosity for the different processing conditions.

The low densities compared with the theoretical one (0.981 g/cm^3^) mainly are due to the trapping of bubbles during the fabrication process. Because of non-simultaneous application of pressure and temperature, polyethylene begins to solidify catching air bubbles. There is an intrinsic porosity in the cardboard before compressing. From the determination of the density, easily measurable, and with the help of the calculated density of the Tetra Brik^®^, the porosity is estimated. Anyway, the best densities are those procured by the combined action of the pressure and the temperature (Figure 7), more concretely in groups G2 and G1, where the maximum pressure and temperature (190 °C and 5 MPa, for 6 and 10 sheets) are combined.

One of the most influent variables is the porosity; in Figure 7, the average values of porosity for the different groups of processing conditions are presented. It affects the strength of the materials. The appearance of pores produces a decrease in flexural strength. It would have to differentiate several porosity types: the total one (it is calculated in this case), the isolated one (it cannot decrease in none of the cases, or in any event it can be create) and the interconnected one (it could fill with a fluid, diminishing the porosity). The grade of porosity of the parts oscillates between 17% and 25%, which is high, because during the fabrication process of the boards, pores are generated in the interior, due to air bubbles being caught by PE during solidification (isolated porosity of new creation). On the other hand, if the pressure application is not uniform in the whole surface, it will also generate porosity. On the contrary, the nearest PE to the cardboard solidifies later and and can infiltrate (filling in of the interconnected porosity). This possible impregnating of PE into cardboard, due to pressure, can suppose a minimization of the porosity and the consistent increase of the density. In general, the density of the boards of 6 sheets (G2, G3, G6 and G7) is higher to those of 10 sheets, because of less risk of porosity creation due to lower contact interfaces during compression. Groups G1 and G2 present the lowest porosity and enhance the average one.

**Figure 8:**
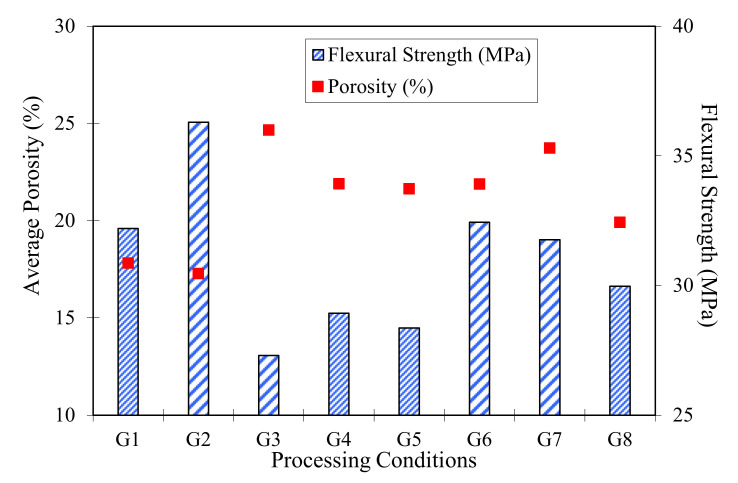
Flexural strength and porosity for the different group of conditions.

On the other hand, in Figure 8, the flexural strength and porosity results for the different families are shown. The fabricated materials with the biggest pressure and temperature presented the maximum values. The porosity influences the flexural strength, and materials with a lower porosity are probably the most reliable. The processing conditions G1 (10 sheets) and G2 (6 sheets) show the best combined values for compression grade, flexural strength, density, and porosity.

The flexural strength of the same waste material (by milling and hot pressing) produces board similar to the wood, but they would be inferior to the same material without milling and with a laminar distribution. This is possible due to a bigger quantity of defects produced during operation of hot pressing, for lack of filling holes between particles of aluminium and cellulose. These holes would possess a fit effect notch in the whole part during a flexural test. The strength is reduced by the porosity and its amount, morphology and distribution. The appearance of these defects in many occasions is due to processing. The influence of these effects is not simple, it is evident that it produces a diminishing of resistant values and will be affected by: size, pore shape, proximity at other defects, its distribution, and the distance between the pores, to the surface or an interface.

**Figure 9:**
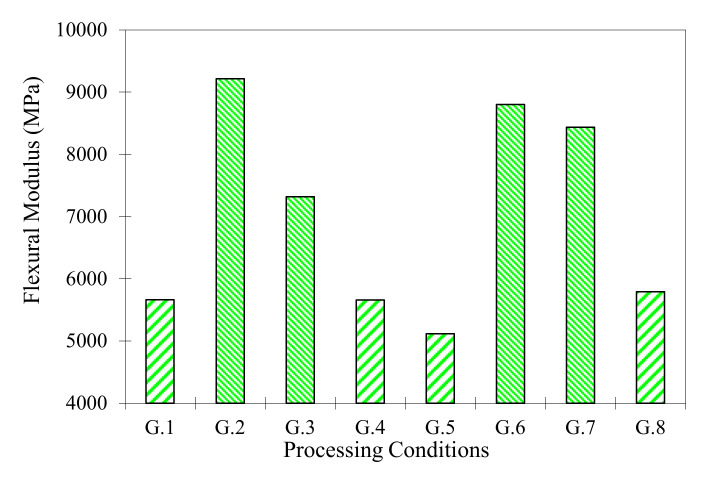
Average flexural modulus for all the series.

In Figure 9, the results for the flexural module are represented. The flexural modulus for the materials under different conditions from the stress-deformation plots was calculated, using the elastic field and quadratic regressions higher than 99%. The values obtained for samples of 10 sheets oscillate between 28.36 and 32.20 MPa for the flexural strength and 5115.9 and 5789.9 MPa for the flexural modulus. Materials with six sheets oscillate between 27.30 and 36.30 MPa for the flexural strength and 7318.0 and 9215.3 MPa for the flexural modulus. The best group is the G2: (T = 190 °C; P = 5 MPa; t = 30 min. and six sheets), with a maximal flexural strength of 36.30 MPa and a flexural modulus of 9215.3 MPa. The flexural strength shows great similarity with laminate boards from high pressure (HPL—High Pressure Laminate) from recycled polymer [51]. These values are high compared with other composite HPL from recycled materials (borax and papers) with higher density [52]. Table 2 shows standard deviations and average values (density, porosity, flexural strength and flexural modulus) for all groups.

In the case of wood for structural use, there are many species and origins, but those that offer similar values of flexural strength to G1 and G2 are from the group of conifers. Some examples are: the Wild Pine (Spain) with values around 30 MPa or Central American Pine species (Pinus Cooperi and Pinus Ponderosa) with values around 35 MPa. [53,54].

Main effects on the flexural strength and flexural modulus are the interaction between the pressure and time, such that higher pressures and maintenance times (in the oven) improve the resistance of the material. The high pressures are able to infiltrate a greater amount of polyethylene in the cardboard, but could cause polyethylene losses, diminishing strength. The temperature (as secondary effect but with great importance) produces the melting of polyethylene more quickly. Improving the values of pressure and oven-time is possible to raise the flexural modulus but avoiding the negative effects that could cause very high values of pressure and time. As a secondary effect, a temperature higher than 190 °C can cause the degradation of the polyethylene and the cardboard.

The elimination of the heat and the maintenance time in the oven have more importance than temperatures (into the studied range 170 to 190 °C). A study [55] with temperatures between 120 and 140 °C and simultaneous application of pressure, shows that the temperature was critical due to being near to the melting point of PE, while the maintenance time passed to be an influence of second order. The values of flexural strength are similar to this study.

Although in this studio and its conditions, the maintenance time is one of the predominant factors, the interaction between the pressure and the temperature is clear because of the results of density and porosity. In such a way, it is possible to melt more polyethylene when increasing the temperature, and when increasing the pressure it is possible to better compress the material, distributing the polyethylene homogeneously for the whole surface and being able to procure a better adhesion in the boards.

Finally, the best groups were chosen to determine the reliability. For the case of 10 sheets (groups G1, G4, G5 and G8), group G1 was elected due it having a bigger flexural strength and density, and lower porosity; and in the case of six sheets (groups G2, G3, G6 and G7), group G2 was chosen, which has the best flexural strength and lower porosity grade. Of each group, 20 samples were manufactured and rehearsed, obtaining the corresponding flexural values and porosity. Four failure estimators were chosen as other studies [45,47]:

(2) $$1\cdot \mathrm{Fj}=\frac{j}{\left(n+1\right)}\;2\cdot \mathrm{Fj}=\frac{j-0.3}{\left(n+0.4\right)}\;3\cdot \mathrm{Fj}=\frac{j-0.5}{n}\;4\cdot \mathrm{Fj}=\frac{j-\frac{3}{8}}{\left(n+1/4\right)}$$

**Figure 10:**
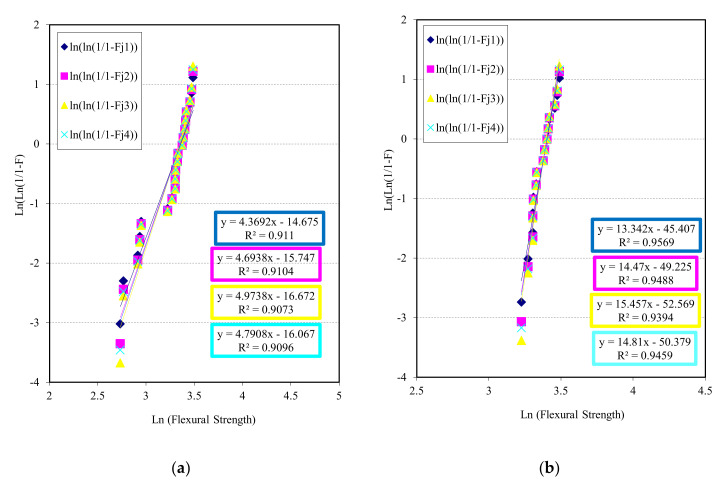
Weibull plots for chosen failure estimators using flexural strength as a key property for tests of conditions G1: (**a**) for all samples and (**b**) eliminated samples with premature fail.

The Fj (failure estimator) is used to calculate the best fit of the linear regression of the scattered points, minimizing the sum of squares of the vertical distances to the regression line. In this way, the slopes of the lines can be calculated, which would give better reliability. Figure 10 shows the results (10a for all samples and 10b eliminated samples with premature fail) for the group of conditions G1 (T = 190 °C; P = 5 MPa; t = 45 min.; and 10 sheets). A good regression adjust can be observed with a value of R2 higher than 0.90 for all estimators (counting all tests) and near 0.95 (eliminating samples with premature fail). Two differentiated fields exist, a first zone where data are grouped with low flexural strength, due to the processing generating areas of weak bonding by polyethylene and those areas causing premature delamination, decreasing the resistant property of the material. An accumulation of porosity (due to variation of the pressure application during processing) can induce premature delamination before higher loads. The failure is more produced due to the higher number of contact interfaces (PE–PE, PE + cardboard–cardboard) than the own porosity. In a second zone, an alignment of data due to very similar flexural strength values is observed. 

Traceability on the different tests has been carried out, so it has been possible to localize and eliminate samples (in representation) that have suffered premature failure (Figure 10a). In this way, it has been possible to verify that those specimens that failed to bond the different sheets have suffered a poor behavior. If all parts were well bonded, a substantial increase in material reliability with respect to flexural strength would be achieved (Figure 10b). Infiltration and bonding between sheets is essential to achieve good mechanical behavior in flexural tests. A combination of pressure, temperature and time, to avoid premature solidification of polyethylene, is necessary.

**Figure 11:**
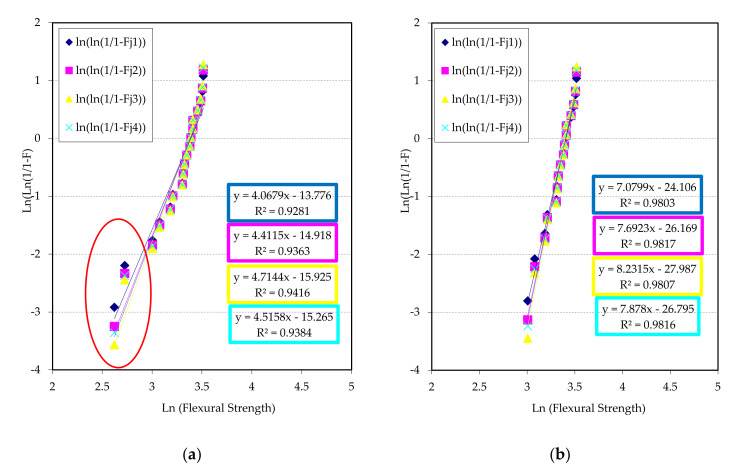
Weibull plots for chosen failure estimators using flexural strength as a key property for tests of conditions G2: (**a**) for all samples and (**b**) eliminated samples with premature fail.

Figure 11 represents the reliable behaviour of material G2 respect flexural strength as key property and the chosen estimators (11a for all samples and 11b eliminated samples with premature fail.). It can be observed in Figure 11a (processing condition G2: T = 190 °C; P = 5 MPa; t = 30 min.; and six sheets), again two differentiated fields exist, a first zone where two data are grouped with low flexural strength, due to the processing generating areas of weak bonding by polyethylene and causing premature delamination, as G1. In a second zone, a better alignment of data is observed. Deleting the two first data, a better correlation and Weibull modulus have been achieved (Figure 11b). A good regression adjust with a value of R2 higher than 0.90 for all estimators (counting all tests) and near 0.98 (eliminating samples with premature fail) can be observed.

Although the regression values are inferior to those of material G1, its adjustments are acceptable. The modules of Weibull are inferior to ones of G1 for the same key property. 

**Figure 12:**
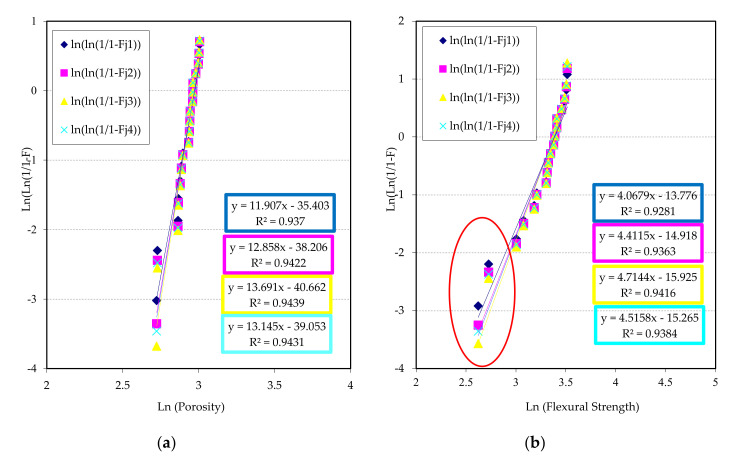
Weibull plots for chosen failure estimators using porosity as a key property: (**a**) G1 series and (**b**) G2 series.

If porosity is used as a key property, the Weibull lines are represented in Figure 12, where the lines of reliability are shown for G1 and G2 series. The Weibull modulus for material G1 is between 11 and 13, being similar to G2 series. In Figure 12a, two well differentiated fields exist, a first zone with a group of five points with low porosity, which could possibly be a polyethylene infiltration, and a second zone where the rest of the data are grouped with more porosity, but next to each other, which contribute to a better adjust and Weibull modulus (R^2^ better than 0.90). Figure 12b presents the line regressions of Weibull corresponding to group G2, taking porosity as a key property. In this occasion, the modules of Weibull are very similar to the previous case, although the dispersion lightly increases to higher than 0.90.

**Figure 13:**
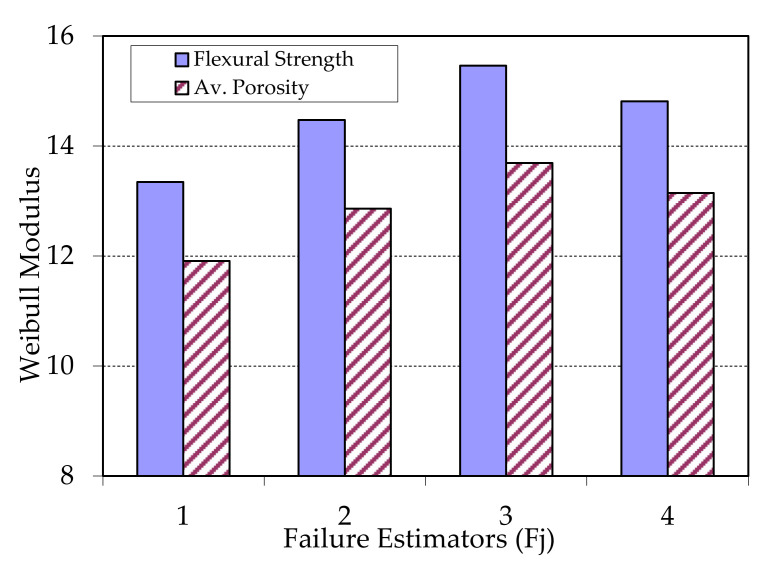
Weibull modulus vs. chosen estimators (Fj = 1 to 4) samples type G1 and for both key properties.

**Figure 14:**
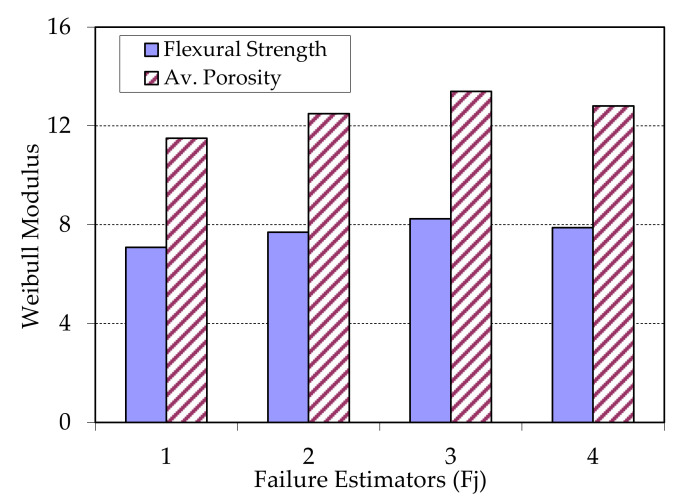
Weibull modulus vs. chosen estimators (Fj = 1 to 4) samples type G2 and for both key properties.

Another bit of information that a Weibull representation can offer is the adjustment of failure estimator comparing with key properties. The estimator determines the separation of the failure function with respect to normal distribution. In Figure 13 and Figure 14, an analysis of the estimators is shown, to evaluate which are the best to determine the reliability of the materials respect key properties (flexural strength and porosity). 

An analysis of Figure 13 concludes that the estimator 3 (Fj = (j−0.5)/n) shows a maximum value for both key properties, with a good quadratic adjustment. In this case, the best key property to determine reliability is flexural strength. This can be due to the combination of high pressure and temperature with a bigger number of sheets in group G1, giving as results some high flexural strength with a bigger amount of polyethylene and therefore a higher probability of adhesion, although also a higher probability of failure due to the isolated porosity. This last reason also contributes to diminish the reliability with respect to porosity. As a conclusion, the measurement of both properties can predict the reliable behaviour of the samples of group G1.

Figure 14 represents that the porosity shows a better key property for the group G2, probably due to the combination of temperature and pressure with a few number of sheets, giving a low number of critical failures. The flexural strength also shows the same trend; although the contribution of adhesion among sheets is lower, lower contact thickness also exists. For both properties, the best failure estimator is the 3 (Fj = (j − 0.5)/n), as in the previous case. The measure of the porosity can give a faithful idea of the reliable behaviour of the samples. The porosity is shown like a suitable key property to evaluate the reliability of these materials, not requiring destructive tests

The election of other estimators could improve the adjustment. Adding to this, the heterogeneity in the pressure application (in fabrication operation) could produce a bigger number of anomalous tests diminishing Weibull modulus. On the other hand, the election of a big size in the samples contributes to raise the probability of premature failures and a great size of defects, diminishing reliability. This analysis can be treated in future investigations.

**Figure 15:**
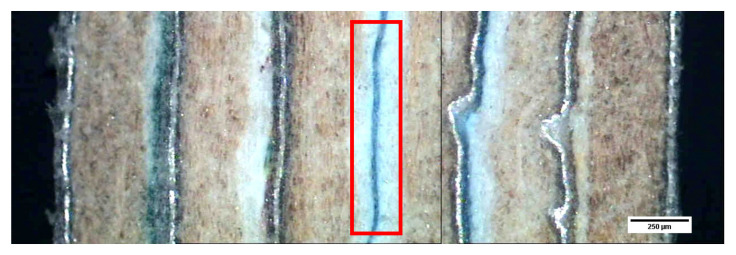
Micrograph of six sheets (G2). The “neutral or central” layer is marked with a rectangle.

**Figure 16:**
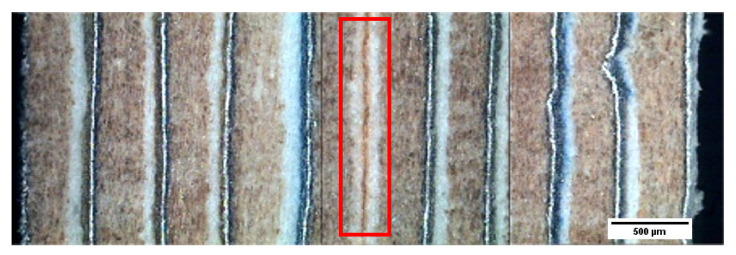
Micrograph of 10 sheets (G1). The “neutral or central” layer is marked with a rectangle.

**Figure 17:**
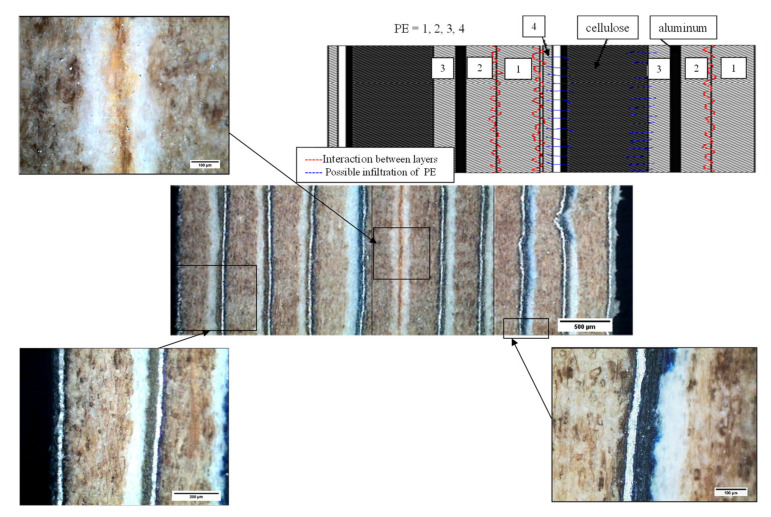
Basic micrograph of 10 sheets (G1). It shows different aleatory zones, and good adhesion between sheets can be observed. In the upper-right part, it shows a general draw of adhesion between two multilayer sheets (or packages).

**Figure 18:**
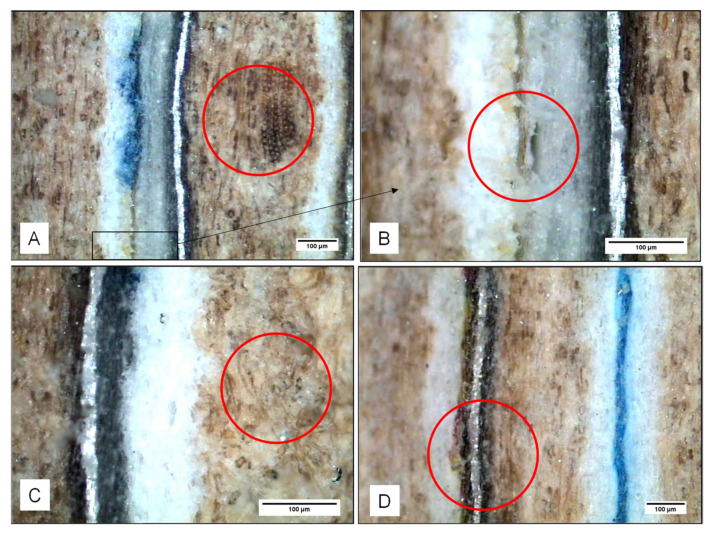
Some of the common failures in the samples before tests: (**A**) and (**B**) lack of adhesion PE–PE, which provokes isolated porosity, (**C**) intrinsic porosity cardboard (cellulose) and (**D**) failure between PE and printing ink.

**Figure 19:**
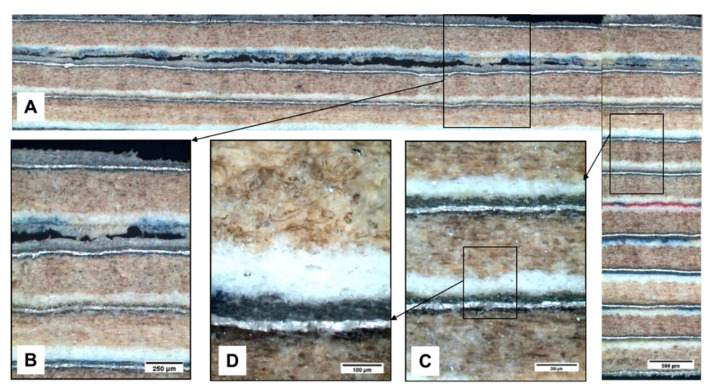
Lack in adhesion post-processing. (**A**) Micrographic mapping longitudinal—transversal of the board. (**B**) Detail area without adhesion PE–PE. (**C**) and (**D**) details into board, where a good binding between sheets is observed.

**Figure 20:**
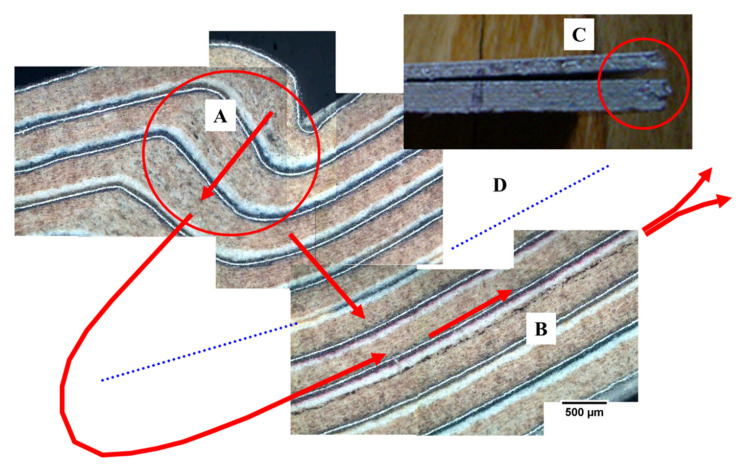
General mechanism of failure in flexion of the multilayer sample of G1 series. (**A**) Generation of a lack of cohesion-defibrillation zone of the cellulose with progression to the centre of the board. (**B**) Lack of cohesion of the interface between PE and cellulose, the weakest zone of the board. The interface PE–PE or neutral layer (**D**) of the centre of the board remains intact (in all the tests) and the stress is transferred to the second or third sheet from surface board. (**C**) Macrograph of the part with the delamination in the third sheet, interface PE–cellulose.

**Figure 21:**
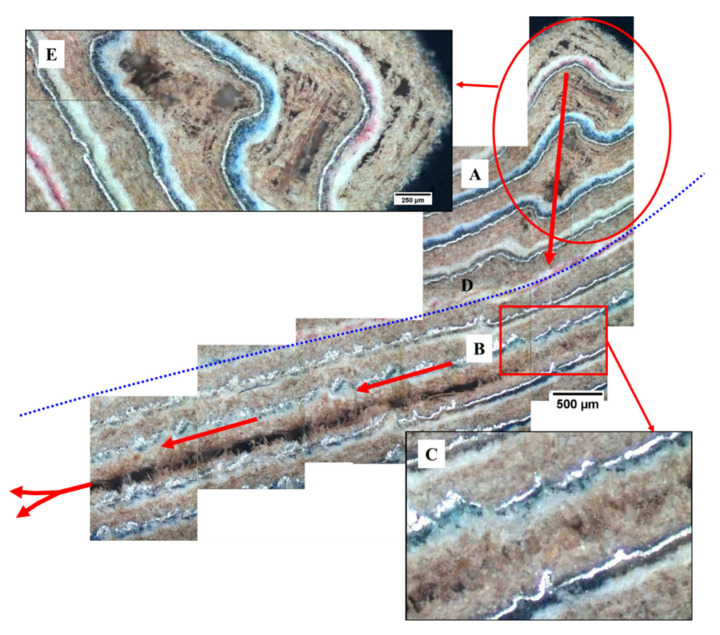
Example of mechanism of failure in the flexion of the multilayer sample of G4 series. (**A**) Generation of a lack of cohesion-defibrillation zone of the cellulose with progression to centre of the board. (**B**) Defibrillation into cellulose, the weakest zone of this type of board. The interface PE–PE or neutral layer (**D**) of the centre of the board remains intact (in all the tests) and the stress is transferred to the second or third sheet from the surface board. (**C**) Detail with the initial defibrillation in the third sheet, into cellulose. (**E**) Detail of the lack of cohesion-defibrillation zone of the cellulose before progression to board centre.

Figure 15, Figure 16, Figure 17, Figure 18, Figure 19, Figure 20 and Figure 21 show a microstructural analysis and more representative failures observed in this type of material. It can make a summary of the different types of failures in features of the different types of porosity and the flexural strength.

Figure 15 and Figure 16 show the general appearance of the board or parts of the G1 and G2 series. In general terms, good adhesion of the different sheets can be seen thanks to the polyethylene that acts as an adhesive at manufacturing temperatures. It should be mentioned that the “neutral or central” layer (double PE–PE, two sheets) will be important when evaluating the flexural behavior of these materials.

The microstructural aspect of the sample of G1 series of 10 sheets (after the compression) is represented in Figure 17. In this figure, different random zones are shown, and a good adhesion between sheets can be observed.

In an approach from the micrographs and the values of flexural strength found for all the test samples, it could be deduced that three intervals of flexural strength exist and therefore three types of predominant failures: failures that cause a low strength with an interconnected porosity (networks of pores, mainly due to the cardboard or the union in the interface PE–PE with a lack of adhesion among the boards); failures that produce parts with a medium flexural strength where the porosity is interconnected and isolated, so much exists cellulose lack of cohesion—defibrillation– as delamination among PE–cardboard; and lastly, failures of high resistance parts due to the isolated porosity (individual pores, enclosed in PE in the interface of union among sheets).

In Figure 18, the most habitual defects are shown (before bending tests); most of the defects are concentrated on the interface PE–PE.

Summarizing, the presence of punctual isolated porosity does not contribute to a great decrease in flexural strength in this type of materials. However, if interconnected porosity appears together with isolated (PE–PE) porosity, this cause big failures, generating premature delamination and reducing the flexural strength considerably. These delaminations are a consequence of a lack of adhesion of PE–PE from compressing operation (Figure 19) and due to the quick heat elimination of the board to compress.

In Figure 20, the general mechanism of failure in flexion for this multilayer material is proposed. When reaching the point of maximum strength, a zone of lack of cohesion, defibrillation of the cellulose is generated with progression toward the centre of the board. The central interface PE–PE of the board is crossed, and the tension is transferred to the second or third sheet (package) counted from the surface. Later, a lack of cohesion and progression of the crack, being localized in the interface between PE and cellulose (in general one of the weakest areas and with a bigger amount of defects), takes place.

Figure 21 shows the phenomenon of lack of cohesion—defibrillation of the cellulose—in a sample of G4 series, where the extension of the defibrillation area focalised in cellulose can be observed. This phenomenon is quite different to lack of cohesion between PE–cellulose. Porosity and density of G4 were high and low, respectively, compared with G1, due to poor infiltration of PE in cellulose. The lack of infiltration makes the zone the weakest when subjected to tension. The neutral layer remains intact like the other parts, so the main difference is the defibrillation of cellulose. The central or “neutral layer” has the particularity that it has four layers of polyethylene, making that area very flexible and transferring the tension to lower layers and sheets.

## 4. Conclusions

It is shown that the alternative of recycling for the multilayer material (pack package), by means of compression with non-simultaneous application of temperature and pressure, is viable. The values of flexural strength are similar or higher to the wood of half strength. 

According to the values of flexural strength of these materials, the product of recycling of flexible packages, and comparing them with the group of wood of half density, a substitution of some of these wood for this waste material seems viable. This solution would avoid its combustion or deposit and could expand the life-cycle of this material. It would be interesting to consider their employment in laminate form and pressed and used before their direct triturating to make panels of agglomerated type. 

A higher compression grade is achieving combining the biggest values of temperature and pressure, producing a volume reduction due to the compression of the cardboard sheet, which possesses a high porosity, and possible infiltration of the polyethylene in the cellulose layer. This phenomenon is more probable in six-sheet parts.

As for the density, the decrease of the real density was observed with respect to the single multilayer sheet, possibly due to the porosity creation in the union PE–PE (because non-simultaneous application of temperature and pressure). As for the porosity, their values are high, mainly due to the time elapsed between the heating operation (oven) and the pressure application, generating bubbles of air, also contributing to the non-homogeneous pressure application on the surface of the boards.

The most influential factors in the process (for the range of chosen conditions) have been the pressure and the maintenance time, the temperature being a second factor. This has been due to parts fabrication by compression (non-simultaneous with temperatures of 170–190 °C) and the temperature is no longer critical compared to maintenance time. 

Low values of bending strenght are produced, due to the premature delamination of the samples during the tests.

Weibull distribution is applicable for these type of parts. As for the Weibull plots (of flexural strength and porosity), two zones are observed. There is a zone with low strengths, due to premature delamination and interconnected porosity that produce big failures. However, Weibull modulus (for both properties) and correlations are good, mainly for G1 series. 

It is not necessary to determine the reliability by means of a destructive test (bending tests), but it is possible through the porosity. This is mainly because the defects are localized preferably in the interface of PE–PE, or due to the intrinsic porosity belonging to the cardboard.

With this, it has been proven that it is possible to replace medium compromise wood with this type of waste in the form of plates or boards, extending its useful life and using it as a multilayer before destroying this geometry.

## Figures and Tables

**Table 1:** Conditions and variables.

Processing Conditions	T (°C)	P (MPa)	t (Min)	e (Sheets)
G1	190	5	45	10
G2	190	5	30	6
G3	190	3	45	6
G4	190	3	30	10
G5	170	5	45	10
G6	170	5	30	6
G7	170	3	45	6
G8	170	3	30	10

**Table 2:** Standard deviations for all groups.

Group	Standard Deviation Density (g/cm^3^)	Standard Deviation Porosity (%)	Standard Deviation Flexural Strength (MPa)	Standard Deviation Flexural Modulus (MPa)
G1	0.01745	1.77915	3.71307	418
G2	0.01413	1.44000	2.35844	528
G3	0.00502	0.51193	3.04083	1367
G4	0.00394	0.40122	1.92001	153
G5	0.01410	1.43683	1.87001	509
G6	0.01242	1.26634	2.98934	357
G7	0.00179	0.18269	1.23030	48
G8	0.01041	1.06129	8.66617	765
